# The complete mitochondrial genome of the Antarctic marbled rockcod, *Notothenia rossii* (Perciformes, Nototheniidae)

**DOI:** 10.1080/23802359.2020.1775507

**Published:** 2020-06-11

**Authors:** Euna Jo, Yll Hwan Cho, Seung Jae Lee, Eunkyung Choi, Jeong-Hoon Kim, Young Min Chi, Jin-Hyoung Kim, Hyun Park

**Affiliations:** a Division of Biotechnology, College of Life Sciences and Biotechnology, Korea University, Seoul, Korea; b Unit of Research for Practical Application, Korea Polar Research Institute (KOPRI), Incheon, Korea; c Division of Polar Life Science, Korea Polar Research Institute, Incheon, Korea

**Keywords:** *Notothenia rossii*, mitochondrial genome, Antarctica, Notothenioidei, PacBio

## Abstract

The complete mitochondrial genome of *Notothenia rossii* was obtained using PacBio Sequel long-read sequencing platform. The mitogenome of *N. rossii* was circular form and 18,274 bp long, which consists of 13 protein-coding genes, 24 tRNAs, 2 rRNAs, and non-coding control region. Particularly, we found duplicated tRNA^Thr^ and tRNA^Pro^ in addition to the typical 22 tRNAs. The phylogenetic tree revealed that *N. rossii* was most closely related to *N. coriiceps* among species in the Nototheniidae clade within the suborder Notothenioidei.

*Notothenia rossii* Richardson, 1844 is a species belonging to the family Nototheniidae, also called the common name marbled rockcod. Like other notothenid fish, *N. rossii* widely distributes in the Southern Ocean and around Antarctica, although the population has been decreased by overfishing due to commercially importance in the fisheries (Barrera-Oro and Marschoff 2007). There are 7 recognized species in the genus *Notothenia* according to the FishBase (Froese and Pauly 2019). Up to now, mitochondrial and whole genome sequences in the genus *Notothenia* have been reported for only one species of *N. coriiceps* (Shin et al. 2014; Oh et al. 2016,). Here, we provide the complete mitochondrial genome of *N. rossii* (GenBank accession No. MT192936) and the results of the phylogenetic analysis with Antarctic fish species.

The sample of *N. rossii* was collected from the sea near Barton Peninsula, King George Island, West Antarctica (62°14′S, 58°47′W). The specimen was deposited at the Earth Biocollection in the Division of Biotechnology, Korea University with accession number KAN0007030. The genomic DNA was extracted by phenol/chloroform method and then g-TUBE (Covaris, CA, USA) shearing device and BluePippin system (Sage Science, MA, USA) were used for preparing 20 kb size-selected templates. The SMRTbell library preparation and sequencing were performed using PacBio Sequel platform according to the manufacturer’s protocol (Pacific Biosciences, CA, USA). The quality and quantity for library were checked with Fragment analyzer (Agilent Technologies, CA, USA) and Qubit 2.0 Fluorometer (Invitrogen, Life Technologies, CA, USA), respectively. PacBio subreads for mitochondrial genome were filtered out using partial sequences of 16 s rRNA and COX1 genes. De novo assembly was conducted by CANU assembler (Koren et al. 2017) and the assembled genome was annotated via MITOS web server (Bernt et al. 2013).

The complete mitochondrial genome of *N. rossii* (GenBank number: MT192936) was 18,274 bp in length, including 13 protein-coding genes, 24 transfer RNA genes (tRNAs), 2 ribosomal RNA genes (rRNAs) and non-coding control region. In particular, duplicated tRNA^Thr^ and tRNA^Pro^ are present in addition to the 22 tRNAs commonly found in fishes. The base composition of A + T (54.10%) was higher than the G + C (45.90%) in the mitogenome. Most of the protein coding genes have typical start codon (ATG) except for COX1, ATP8, and ATP6 genes (GTG). Three types of stop codons (TAA, TAG, and T(AA)) were identified. The phylogenetic relationships of *N. rossii* were analyzed with 10 Antarctic species in the suborder Notothenioidei using 13 protein-coding genes (Figure 1). Maximum Likelihood (ML) tree was built with 500 bootstrap replications and JTT matrix-based model (Jones et al. 1992) by MEGA X software (Kumar et al. 2018). The tree showed that *N. rossii* was first clustered with *N. coriiceps*, and they were grouped with species belonging to Nototheniidae (*Dissostichus eleginoides*, *D. mawsoni*, *Pagothenia borchgrevinki* and *Pleuragramma antarcticum*). This result provides fundamental data to further our understanding of the evolutional relationship of Antarctic fishes in the Southern ocean.

**Figure 1.**
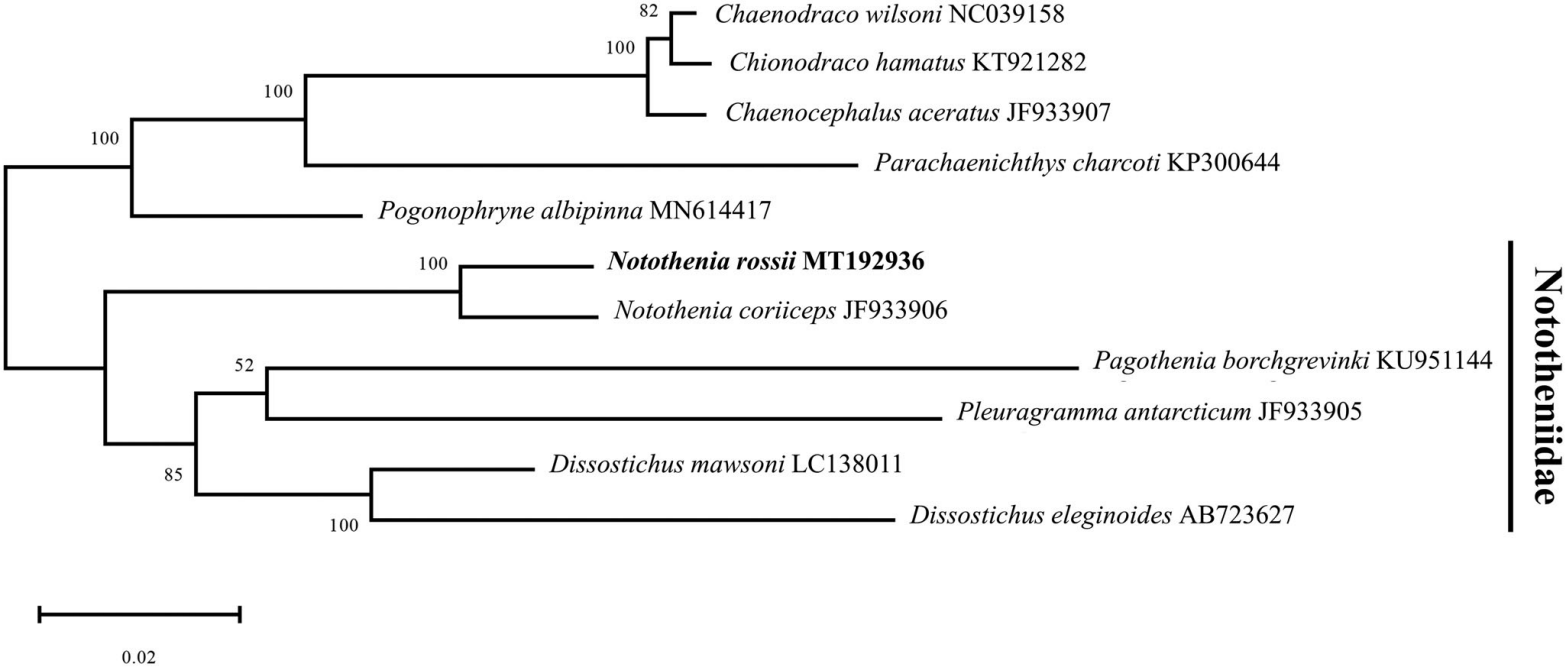
Maximum likelihood (ML) tree for *Notothenia rossii* within the suborder Notothenioidei. The scientific name and GenBank accession number were shown for each species.

## Data Availability

The data that support the findings of this study are openly available in NCBI under the accession MT192936 (https://www.ncbi.nlm.nih.gov/nuccore/MT192936.1/).
